# Homoplantaginin exerts therapeutic effects on intervertebral disc degeneration by alleviating TNF-α-induced nucleus pulposus cell senescence and inflammation

**DOI:** 10.3389/fphar.2025.1526107

**Published:** 2025-03-27

**Authors:** Jiadong Guo, Pu Zhang, Xiao Yang, Xin Wang, Xiankun Cao, Jie Zhao

**Affiliations:** Shanghai Key Laboratory of Orthopedic Implants, Shanghai Ninth People’s Hospital, School of Medicine, Shanghai Jiaotong University, Shanghai, China

**Keywords:** inflammation, senescence, nucleus pulposus cell, intervertebral disc degeneration, homoplantaginin

## Abstract

The incidence of intervertebral disc degeneration (IDD) is increasing year by year, while the age of onset of IDD is decreasing year by year. For the individuals affected by IDD, an alternative treatment to surgery is required. IDD is thought to be related to nucleus pulposus (NP) cell senescence and inflammation. Therefore, inhibition of NP cell inflammation and senescence may counteract IDD. We screened 20 small-molecule drugs to compare their anti-inflammatory effects, and finally selected homoplantaginin (HPG) as a treatment regimen. HPG is an extract of *Salvia miltiorrhiza* with anti-inflammatory properties. The objective of this research was to investigate if HPG could have a therapeutic effect on IDD through its anti-inflammatory or anti-aging effects. We identified the appropriate concentration of HPG in primary NP cell cultures and demonstrated that it inhibited inflammatory pathway activation and reduced the senescence phenotype of NP cells *in vitro*. And *in vivo*, the therapeutic effect of HPG on caudal disc degeneration was confirmed consistently. In conclusion, our findings suggest that HPG can alleviate the degeneration in the pathogenesis of IDD and that it has a potential effect in the treatment of IDD.

## 1 Introduction

Lumbar intervertebral disc degeneration (IDD) is one of the most important potential cause of lower back pain, the primary cause of labor loss (Buchbinder et al., 2018). At present, the incidence of IDD is increasing year by year, causing great pain to patients and greatly increasing the economic burden on society (Andersson, 1999). More importantly, the average age of onset of lumbar disc degeneration has decreased compared to that in the past (Lippross et al., 2021). In younger patients, surgery is not the first option. Thus, there is an urgent need to identify a suitable alternative treatment for lumbar intervertebral disc surgery (Hartvigsen et al., 2018).

The lumbar disc is composed of the nucleus pulposus and annulus fibrosus. Owing to aging, trauma, genetics, and other factors, the lumbar intervertebral disc exhibits annular fibrosis narrowing and even rupture, dehydration and shrinkage of the NP, loss of intervertebral height, vertebral instability, and other pathological changes, leading to lumbar IDD (Bonnevie et al., 2019). NP cells are the main functional cells located in the nucleus pulposus that secrete extracellular matrix (ECM). NP cells are cartilaginous cells that are present in the nucleus pulposus of the disc in adults, replacing the chordal cells in childhood. Similar to chondrocytes, NP cells are fusiform or polygonal in shape. Current research suggests that disc degeneration does not have much to do with notochord cells because they are lost and replaced by nucleus pulposus cells in adult discs. Accordingly, the cell secretion function, senescence and degeneration of nucleus pulposus are more related to the senescence of NP tissue and the occurrence of IDD. The main function of NP cells is to secrete ECM (Roberts et al., 2006).

As early as 1934, Mixter and other scientists proposed that a herniated disc compresses the dural sac and directly compresses and irritates nerve roots, causing sciatica symptoms in patients. Since then, the theory of pain caused by simple mechanical compression has been widely accepted and spread. However, with the development of time and the progress of time, more and more clinical evidence shows that mechanical compression pain alone cannot explain and include the degree of disc degeneration and clinical manifestations. Clinical data show that some patients have obvious disc herniation but no typical neurological symptoms, and a considerable number of patients have no obvious disc herniation but show strong sciatica symptoms, even more serious than the symptoms of disc herniation with clear imaging findings. These findings have raised questions about the theory of pain caused by simple mechanical compression. On the other hand, edema and congestion of nerve roots can be observed in most patients with severe neurological symptoms. These clinical phenomena seem to indicate that IDD is considered relative to oxidative stress such as inflammation.

Since the 2000s, researchers have gradually discovered a link between inflammation and many diseases. The occurrence of inflammation cannot be separated from the activation of inflammatory pathways. Currently, it is generally accepted that after receiving external oxidative stimulation, inflammatory factors such as TNF-α, IL-6, and IL-1β are upregulated and released, bind to membrane receptors on the surface of cell membranes, and transmit signals. It can induce phosphorylation and activation of downstream inflammatory pathways such as MAPK, NF-κB, PI3K/AKT, JAK/STAT, and then form corresponding complexes with downstream related proteins through cascade reactions, and finally enter the nucleus through the nuclear pore, bind to intracellular receptors, and regulate transcription and translation of related gene proteins. In nucleus pulposus cells, inflammatory factors activate inflammatory pathways, enter the nucleus after a series of reactions, and exert regulatory effects on matrix metalloproteinases (MMP3, MMP9, MMP13) associated with extracellular matrix catabolism in nucleus pulposus and proteoglycan and collagen (AGREECAN, COLII) associated with anabolism. Eventually, the imbalance between catabolism and anabolism of the extracellular matrix in the nucleus pulposus would result in the influence of composition and volume of contents in the NP, dehydration and shrinkage of NP, and eventually intervertebral disc degeneration. TNF-α is a crucial factor involved in inducing senescence and inflammation in NP cells (Novais et al., 2021). It can activate classical inflammatory pathways such as MAPK and NF-κB and influence downstream catabolism and anabolism in NP cells (Che et al., 2020).

TNF-α is a crucial cell factor in inducing senescence and inflammation in NP cells. It can activate classical inflammatory pathways such as MAPK and NF-κB and affect downstream catabolism and anabolism in NP cells. In recent studies, TNF-α has been reported to induce inflammatory responses in NP cells through MAPK and NF-κB pathways. Stimulation of nucleus pulposus cells with TNF-α resulted in phosphorylation of the MAPK and NF-κB pathways in NP cells, increased expression of downstream matrix metalloproteinase MMP series proteins associated with extracellular matrix decomposition in NP, and decreased expression of AGREECAN associated with extracellular matrix synthesis in NP. In addition, the downstream of the MAPK pathway also includes P53, P21, and other proteins related to cell aging. Phosphorylation and activation of the MAPK pathway will increase the expression of cell aging-related proteins and thus lead to the increase of the nucleus pulposus cell aging phenotype. Thus, inhibition of TNF-α-induced inflammatory pathway activation may inhibit the progression of disc degeneration by inhibiting inflammation and senescence in nucleus pulposus cells (Johnson et al., 2015). Therefore, inhibition of TNF-α, and thereby, inhibition of NP cell senescence and inflammation, may be the direction for an alternative therapy for IDD.

In the present research, we found that TNF-α induced NP cell inflammation mainly via the MAPK and NF-κB pathways. Phosphorylation of these pathways leads to increased downstream expression of matrix metalloproteinase (MMP) and increased decomposition of ECM in NP cells. It has been reported that inhibition of TNF-α-induced phosphorylation of these two pathways can have a therapeutic effect on disc degeneration. In addition, TNF-α can induce cell senescence, while the aging of NP cells is also considered to be an essential mechanism of intervertebral disc degeneration. Homoplantaginin (HPG) was selected after we tested 20 small-molecule drugs for their anti-inflammatory ability. HPG is the main flavonoid in the Chinese herb *Salvia miltiorrhiza*, and has anti-inflammatory and antioxidant effects (Xu et al., 2018). HPG has previously been shown to inhibit phosphorylation of NF-κB pathway and was used to suppress liver inflammation (Wu et al., 2012). In this study, HPG inhibited NP cell senescence and inflammation *in vitro*, and its therapeutic effect on IDD was further confirmed by local injection in a rat model of coccygeal puncture.

## 2 Materials and methods

### 2.1 Chemicals and reagents

Homoplantaginin was obtained from Selleck Chemicals (Houston, TX, United States), and was dissolved in dimethyl sulfoxide (DMSO) and stored at −20°C.

Before being used on cells, it was diluted in cell culture medium until the final concentration of DMSO was less than 0.1%. Dulbecco’s modified Eagle’s medium (DMEM), 0.25% trypsin EDTA solution, fetal bovine serum (FBS), and 1% penicillin–streptomycin mixture were obtained from Gibco (Thermo Fisher Scientific, Waltham, MA, United States). FBS was obtained from GIBCO BRL (Sydney, Australia). Insulin Transferrin Selenium (ITS) reagent and Alcian blue (AB) solution were obtained from Sigma-Aldrich (St. Louis, MO, United States). The Prime Script Reverse transcription Kit together with TB-Green Premix Ex Taq Kit were both obtained from Takara Biotechnology (Shiga, Japan). TNF-α was purchased from R&D Systems (Minneapolis, MN), while phosphatase and protease inhibitors, together with RIPA lysis buffer were obtained from Roche (Basel, Switzerland). The Cell Counting Kit-8 (CCK-8) was obtained from Sangong Biotechnology Co. Ltd. (Shanghai, China). Involved primary antibodies and secondary antibodies were all purchased from Cell Signaling Technology (CST, Danvers, MA, United States).

### 2.2 Cell counting kit-8 assay

Cell proliferation was measured using the CCK-8 kit. In order to clear and define the influence of HPG on cell proliferation, NP cells were inoculated into 96-well plates, amd the density of NP cells was 7,000 cells per well. Then, HPG was added at grading concentrations (0, 1, 5, 10, 15, or 20 μg/mL), and cultured together for 12, 24, 48, or 72 h. When incubated until the specified time, the NP cells were cultured with 100 µL complete media which contained 10 μL of CCK-8 reagent at 37°C. 2 h ago, use a spectrophotometer, which was the components of an Infinite M200 Pro multimode microplate reader, to record the optical density (OD) values of those cells at 450 nm.

### 2.3 Primary NP cell isolation and culture

Sprague-Dawley (SD) Rats used in this study were provided from Shanghai Lab, Animal Research Center, Shanghai, China. After the rats were killed by CO_2_ euthanasia when they were 6 weeks old, their tails were cut and disinfected through medical alcohol for half an hour. The skins on the tails were removed to expose the discs. Then, the cartilaginous endplate was incised separately, and the NP was removed and digested with 1% type II collagenase for 2 h in a CO_2_ incubator at 37°C. After suspension centrifugation, cell pellet was resuspended and then planted in a culture dish with DMEM (1% penicillin–streptomycin, 10% FBS). During the whole culture process, involved cells were all cultured at 37°C, 5% CO_2_, and 21% O_2_.

### 2.4 Western blot

RIPA lysis buffer mixed with phosphatase and protease inhibitors was used to extract proteins. A BCA protein quantitative kit was used to quantify the concentration of those extracted proteins. 20 μg of the proteins were separated through 4%–20% SDS-PAGE gels, followed by electroblotting on 0.22 µm PVDF membranes. The membranes were washed by TBST buffer thrice for a quarter of an hour each time after blocking with 5% BSA-TBST for 1 h at room temperature and incubated with the related primary antibodies (diluted 1:1,000) overnight at 4°C. And then, after membranes were washed with TBST thrice for 15 min each time, they were incubated with corresponding anti-mouse or anti-rabbit secondary antibodies (diluted 1:50,00) for 1 h in the dark environment at RT. Then, TBST was used to wash the membranes thrice for 10 min each time once more. In the end, immunoblotting was displayed by the Odyssey infrared imaging system (LI-COR, Lincoln, NE, United States). The protein bands were analysed by the ImageJ software (National Institutes of Health, United States).

### 2.5 High density culture and AB staining

In order to assess the effects on cell differentiation, high density cultured NP cells was examined with AB staining. Firstly, NP cells were planted in 24-well plate with 10 μL per well at a density of 1.0 × 10^7^ cells/mL. Then those plates were placed in CO_2_ incubator at 37°C. After 2 h, every well was added with 1 mL of DMEM. In the next day, medium in each well was changed to 1 mL of DMEM, contained with ITS (10 ng/mL), TNF-α (10 ng/mL), or HPG (20 μg/mL) with TNF-α (10 ng/mL). The medium was refreshed every 3 days until 7 days. Then those NP cell clusters were stained by AB solution at RT overnight. After washing out the dye, images were captured using Leica DM4000 B microscope, and the IOD was evaluated using ImageJ.

### 2.6 Quantitative real-time polymerase chain reaction analysis

First, NP cells handled with corresponding treatments were planted in 6-well plates and incubated in 37°C CO_2_ incubator for 24 h. In accordance with the manufacturer’s protocol, Total RNA was isolated by TRIzol reagent. The extracted RNA reverse-transcribe to cDNA through a cDNA synthesis kit. Quantitative Real-Time PCR was performed on a PCR system. The expression of involved mRNA was statistical analysed by the 2^−ΔΔCT^ method using β-actin as an internal reference. Specific primer pairs were designed using NCBI BLAST (Table 1).

**TABLE 1:** PCR primers information.

Gene	Forward primer	Reverse primer
B-actin	CCC​GCG​AGT​ACA​ACC​TTC​T	ATG​CCG​TGT​TCA​ATG​GGG​TA
MMP3	CCT​CTG​AGT​CTT​TTC​ATG​GA	TGT​CTG​TAG​CCC​AGG​AGT​GT
MMP9	ATG​GTG​CCC​CAT​GTC​ACT​TT	CAC​CAG​CGA​TAA​CCA​TCC​GA
MMP13	CCA​ACC​CTA​AGC​ACC​CCA​AA	TCT​CGG​GAT​GGA​TGC​TCG​TA
SOX9	TCC​CCG​CAA​CAG​ATC​TCC​TA	AGC​TGT​GTG​TAG​ACG​GGT​TG
Aggrecan	GCT​ACC​CTG​ATC​CCT​CAT​CC	GAT​GTC​CTC​TTC​ACC​ACC​CA
P53	ATG​GAG​GAG​CCG​CAG​TCA​G	TCA​GTC​TGA​GTC​AGG​CCC​TTC
P21	CAG​ACA​TTC​AGA​GCC​ACA​GG	CTA​AGG​GCC​CTA​CCG​TCC​TA

### 2.7 Senescence assays

The degree of senescence in primary NP cells was analyzed through aβ-galactosidase staining kit, according to the manufacturer’s instructions. NP cells were planted into a 24-well plate at a density of 30,000 cells per well, and incubated with TNF-α at the concentration of 10 ng/mL, with or without HPG (20 μg/mL) for 3 days. Cells were immobilized at RT for 15 min with 1X fixation solution, then cultured overnight with the β-Gal staining solution in a CO_2_-free incubator. After washing with alcohol, cells were photographed under a light microscope. ImageJ was used to measure SA-β-Gal–positive cells.

### 2.8 Immunofluorescence

First, NP cells were planted in 35 mm Nunc™ glass Petri dishes at a density of 40,000 cells per well. Second, they were incubated with TNF-α (10 ng/mL) with or without HPG (20 μg/mL) for 1 day. After washed with PBS thrice, 4% paraformaldehyde was used to fix cells for 40 min. Subsequently, those NP cells were infiltrated by 0.5% Triton X-100 at RT for 10 min and blocked with 5% BSA for 1 h after three more washes. Then, the NP cells were incubated using the primary antibody under 4°C all-night. In the next day, PBS was used to wash NP cells thrice more, and then incubated with related secondary antibody (diluted 1:1,000) for 1 h, and next stained with DAPI for 5 min in the dark environment. Digital fluorescence images were performd through a laser scanning microscope. In the end, The IOD of the target gene and DAPI was determined for semiquantitative analysis.

### 2.9 Animals and surgical procedures

Twelve 14 weeks old male SD rats were reared in a sterile environment at a suitable temperature and humidity. Food and water were provided. Caudal puncture was used to build the model of IDD in SD rat. Shortly, before surgery, pentobarbital was injected intraperitoneally to anesthetize the rats and then medical alcohol used to disinfect their tails. Their Co7/8 and Co8/9 discs were then punctured through the dorsal skin using a sterile 20-gauge needle to the center of the NP, rotated 360°, and held for 1 min. Finally, after the needle was removed, the skin of the tail was disinfected again to avoid infection. Local injection of 50 μL HPG (50 μg/mL) through the Co8/9 disc of the tail vertebra was performed weekly with a microsyringe. Simultaneously, the Co7/8 disc was weekly administered normal saline (the same volume as the treatment group). A control group of Co6/7 discs, without surgery or treatment, was selected. The rats were sampled 1 month later.

### 2.10 X-ray analysis

The tail of each rat was photographed and analyzed using X-ray. In the anteroposterior axis, digital imaging was captured using a 21 lp/mm detector that provided up to ×4 geometric magnification. The disc height index (DHIs) was computed and the formula is as follows: DHI = Intervertebral disc (IVD) height/adjacent IVD body height.

### 2.11 Magnetic resonance imaging

The rat tail was photographed and analyzed using MRI. Rat caudal disc signals were obtained on sagittal T2-weighted images (T2WI) using a 1.5T MRI (Philips Eclipse, Cleveland, OH, United States). The intervertebral disc moisture content and degree of degeneration were evaluated based on the intervertebral disc T2 signals.

### 2.12 Histological analysis

Tail IVD tissue was separated and fixed in paraformaldehyde (4%) for no less than 48 h. The sample was then planted in paraffin wax. Then a continuous section of 5 μm was obtained in the middle of the sagittal plane. The structural changes in the IVD were then observed by staining with saffron O-fast green (SO-FG), hematoxylin-eosin (HE). The histological scores were calculated using a modified histological grading system.

### 2.13 Immunohistochemistry

First, the previous sections were successively dewaxed, dehydrated. Later, 3% H_2_O_2_ used to treat sections about 12 min at RT, and then 1% goat serum used to block those sections at RT for 1 h. After, primary antibody was used to incubate sections at 4°C overnight. In the next day, those sections were rinsed twice by PBS and incubated with enzyme-conjugate secondary antibody at RT for 2 h. A Leica DM4000B microscope was used to obtain digital images. The IOD was analyzed using PP6.0.

### 2.14 Statistical analysis

All the involved experiments were conducted no less than thrice to obtain the data. And all of the data are expressed as the mean ± standard error of the mean. The GraphPad Prism 9.0 Software (GraphPad Software, San Diego, CA, United States) was used to measure those significant differences among study groups. The differences between the groups was compared by One-way analysis of variance (ANOVA). A p value < 0.05 was regarded statistically significant.

## 3 Results

### 3.1 Anti-inflammatory drug screening

**FIGURE 1:**
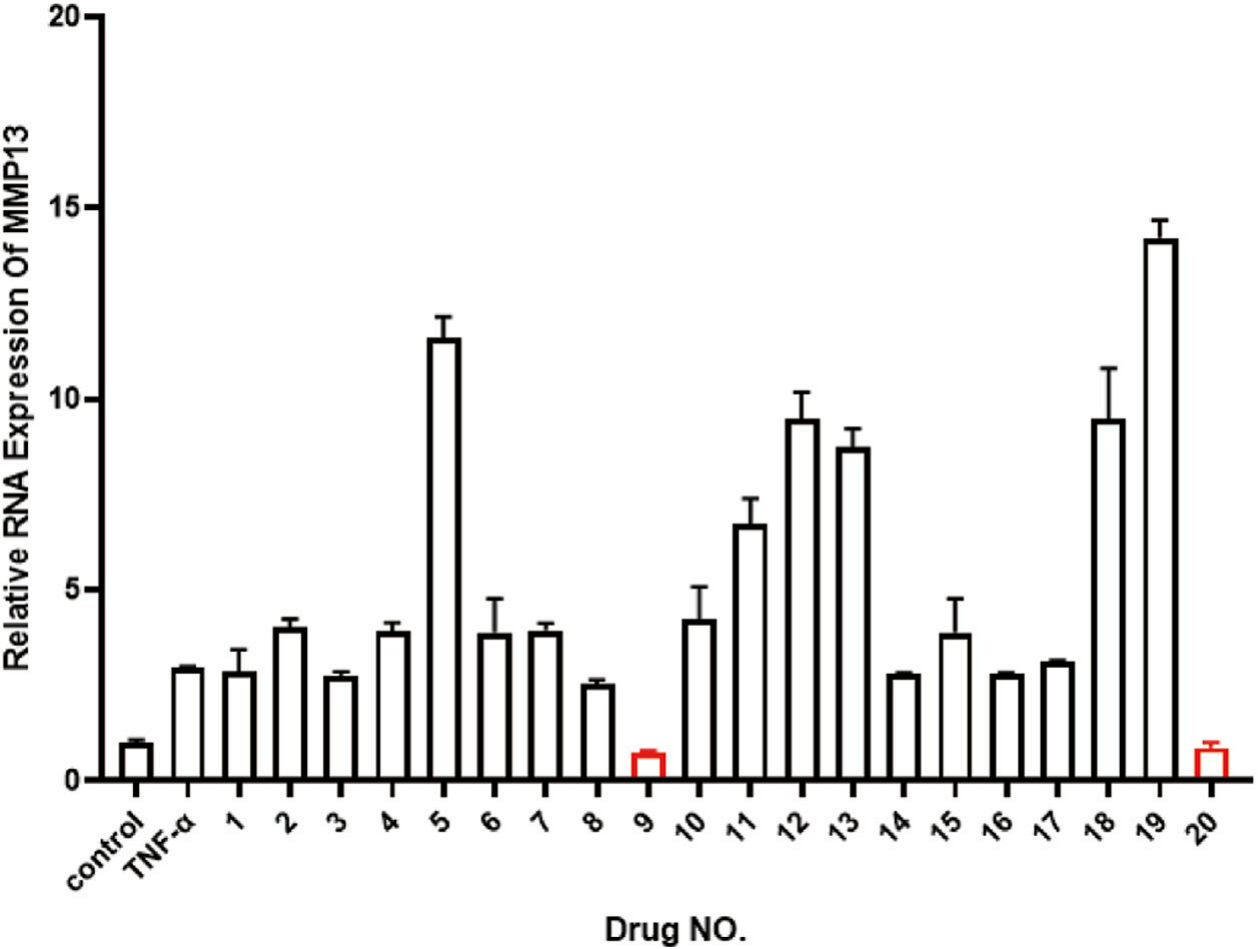
Real-time quantitative polymerase chain reaction (RT-qPCR) was used to detect the expression of MMP13 after TNF-α treatment to compare 20 small molecule drugs. ALL data are presented as the mean ± SEM of three independent experiments (n = 3. **p < 0.01, ***p < 0.001 and ****p < 0.0001).

In order to find suitable drugs to combat TNF-α induced inflammatory pathway activation. We selected 20 small molecule compounds and applied them to nucleus pulposus cells according to the experimental concentration provided by the manufacturer (Table 2). After 24 h of co-culture with TNF-α, qPCR was performed to compare their effects on matrix metalloproteinase MMP downstream of the inflammatory pathway of nucleus pulposus cells. After analyzing the effects of 20 small molecule compounds, we selected homoplataginin, which Cas number is 17680-84-1 and has significant anti-TNF-α effect. By comparison, we found that the expression of MMP13 protein was upregulated 3 times under the action of TNF-α, but HPG could significantly reverse the effect of TNF-α. In addition, another small molecule KIRA6 showed a slightly lower inhibitory effect on TNF-α than HPG. To further explore the role of small molecule compounds in inhibiting intervertebral disc degeneration by inhibiting TNF-α-induced inflammatory pathway activation in NP cells, we chose HPG, which has a more obvious effect, as the main research object (Figure 1).

**TABLE 2:** The Cas numbers of 20 small molecule compounds.

NO.	Drug	Cas	NO.	Drug	Cas
1	Monocrotaline	315-22-0	11	(S)-Trolox	53174-06-4
2	Cytisine	485-35-8	12	Picroside II	39012-20-9
3	Quinine	130-95-0	13	Echinocystic acid	510-30-5
4	Quinidine	56-54-2	14	Propyl gallate	121-79-9
5	Artesunate	88495-63-0	15	Myricitrin	17912-87-7
6	Myricitrin	17912-87-7	16	Propyl gallate	121-79-9
7	Rapamycin	53123-88-9	17	5,7-Dihydroxychromone	31721-94-5
8	Camptothecin	7689-03-4	18	Jatrorrhizine hydroxide	483-43-2
9	Homoplantaginin	17680-84-1	19	Oridonin	28957-04-2
10	Bisdemethoxycucurmin	33171-05-0	20	kira6	1589527-65-0

### 3.2 Effect of HPG on cell proliferation

**FIGURE 2:**
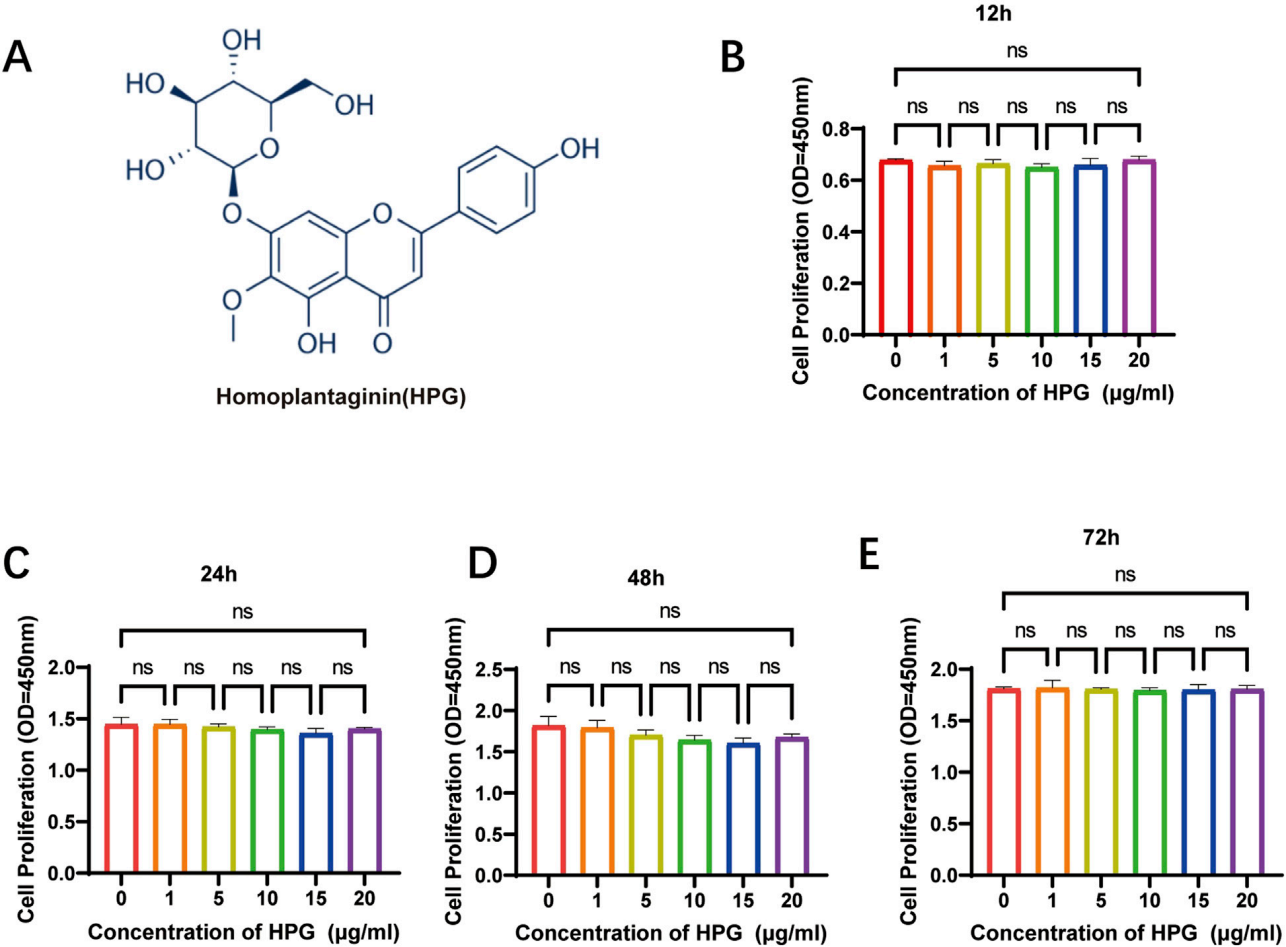
Effects of Homoplantaginin (HPG) on proliferation of nucleus pulposus (NP) cells. **(A)** The chemical structure of HPG **(B–E)** Effects of HPG with different concentration on proliferation of nucleus pulposus. NP cells were inoculated at 12, 24, 48, or 72 h. The inhibitory effect of HPG on the proliferation of NP cells was detected using the CCK-8 assay. Data are presented as the mean ± SEM (n = 3).

In Figure 2A the chemical structure of HPG is shown. The effects of HPG (1 mg dissolved in 50 μL DMSO) on cell proliferation were determined using HPG gradient concentrations of 0, 1, 5, 10, 15, or 20 μg/mL according to the maximum toxicity range allowed by DMSO. For proliferation assays, 3,000 NP cells per well were planted in 96-well plates and cultured in complete DMEM in the presence of 0, 1, 5, 10, 15, or 20 μg/mL of HPG. To explore the effect of HPG on the proliferation of NP cells, NP cells were inoculated at 12, 24, 48, or 72 h, and the inhibitory effect of HPG on the proliferation of NP cells was detected using the CCK-8 assay. We observed that with the increase of stimulation concentration and time, HPG did not lead to a significant increase in OD value of NP cells. The results showed that HPG had no obvious inhibitory effect on NP cell proliferation within the maximum toxicity range allowed by DMSO (Figures 2B–E).

Based on the result that HPG had no obvious toxic effect on the proliferation of NP cells, the maximum non-toxic HPG concentration allowed by DMSO (20 μg/mL) was selected for the following study.

### 3.3 HPG inhibits the activation of inflammatory pathways

**FIGURE 3:**
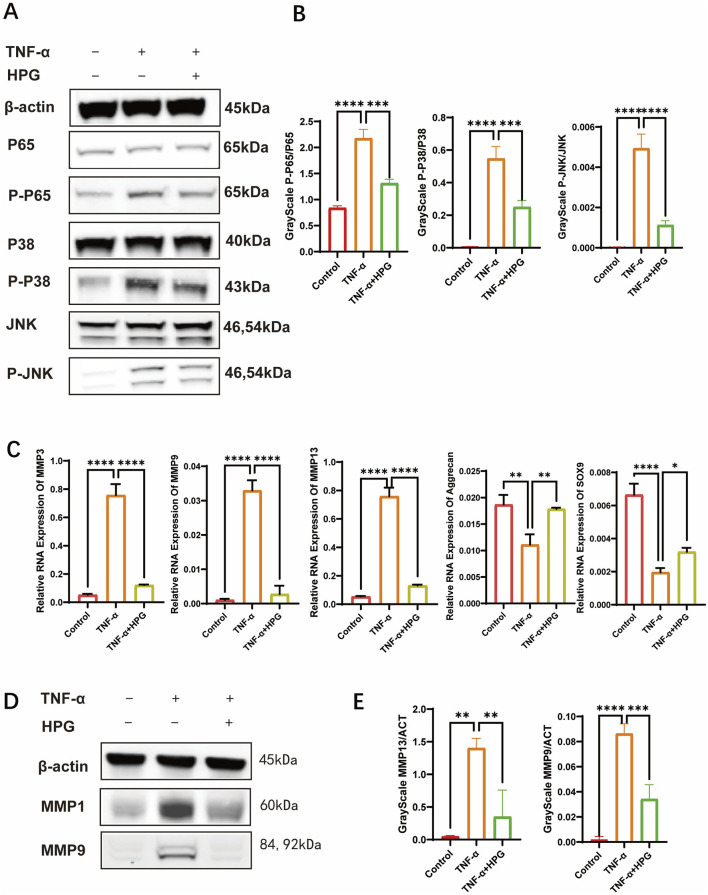
Homoplantaginin (HPG) inhibited TNF-α-induced inflammatory pathway activation and influenced subsequent and NFκB inflammatory pathways in TNF-α-induced NP cells **(A)** The results of western blot ECM decomposition and anabolism **(B)** Western blotting showed that HPG inhibited the activation of MAPK were analyzed by gray ratio analysis. Calculate the gray scale ratio of phosphorylated and non-phosphorylated proteins. ALL data are presented as the mean ± SEM (n = 3. ***p < 0.001 and ****p < 0.0001) **(C)** The quantitative real-time polymerase chain reaction (RT-qPCR) was performed to detect the expression of the anabolism and catabolism genes after treatment with TNF-α (20 ng/ml) with or without HPG (20 μg/ml) for 24 h. The expression of those genes was normalized to the expression of β-actin (MMP3, MMP9, MMP13, SOX9, Aggrecan). ALL data are presented as the mean ± SEM of three independent experiments (n = 3. ***p < 0.001 and ****p < 0.0001) **(D)** Western blotting showed that HPG inhibited the expression of MMP9 and MMP13 in TNF-α-induced NP cells. **(E)** The results of western blot were analyzed by gray ratio analysis. Calculate the gray scale ratio with β-actin. ALL data are presented as the mean ± SEM (n = 3. ***p < 0.001 and ****p < 0.0001).

To investigate the effect of HPG on the activation of inflammatory pathways in NP cells, NP cells were pretreated with 50 μg/mL HPG for 2 h and stimulated with TNF-α for 15 min to induce the activation of inflammatory pathways. Subsequent Western blot showed that TNF-α stimulation upregulated the phosphorylation of P65, P38, JNK, and other inflammatory pathway-related proteins in NP cells significantly. However, phosphorylation of these proteins was inhibited in NP cells treated with HPG and stimulated with HPG. Our results suggested that HPG can inhibit the activation of the MAPK and NF-κB pathways (Figures 3A, B).

### 3.4 HPG inhibited the activation of inflammatory pathways

**FIGURE 4:**
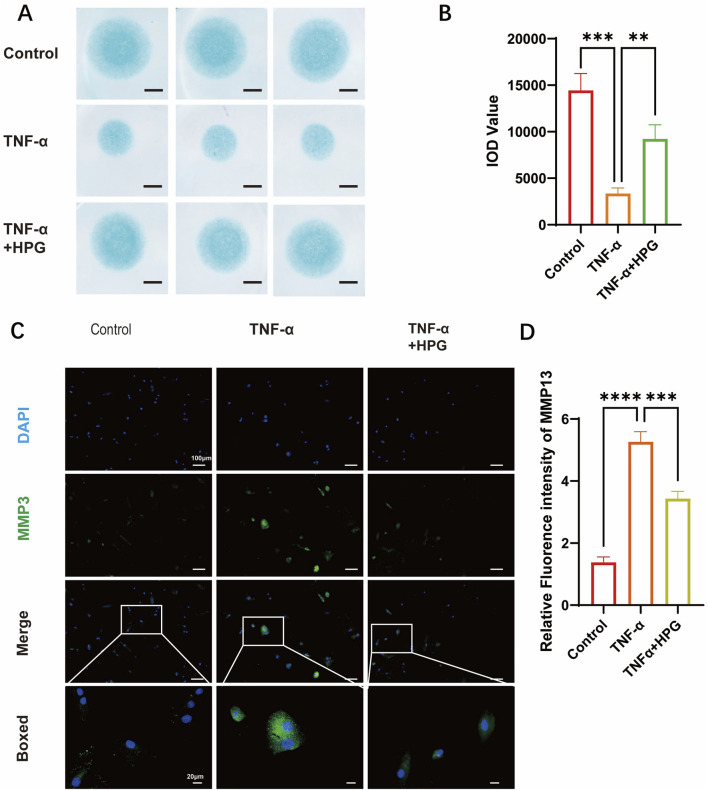
Homoplantaginin (HPG) inhibited TNF-α-induced extracellular matrix (ECM) degradation **(A)** Alcian blue results of NP primary cells (P3) on high-density culture. Scale bar, 1mm **(B)** The integrated optical density (IOD) value of Alcian blue were performed to evaluate the extracellular matrix (ECM) of NP primary cells (P3). ALL data are presented as the mean ± SEM (n = 3. ***p < 0.001 and ****p < 0.0001) **(C)** Immunofluorescence result of MMP13 after NP cells treated with or without TNF-α and HPG. Scale bar, 10 μm **(D)** Fluorescence intensity analysis were peformed to evaluate the expression of MMP13. ALL data are presented as the mean ± SEM (n = 3. ***p < 0.001 and ****p < 0.0001).

Real-Time qPCR was used to detect the effect of HPG on the transcription of genes related to ECM anabolism and catabolism. The results showed that the expression of MMP3, MMP9, and MMP13, which are associated with increased ECM catabolism, was upregulated after TNF-α treatment, and the addition of HPG significantly reversed this effect. Furthermore, the mRNA expression of sox9 and aggrecan, which are related to ECM anabolism, was decreased after TNF-α stimulation, and as expected, the combined use of HPG inhibited the anabolic inhibitory effect of TNF-α (Figure 3C). Consistently, Western blotting results showed that TNF-α treatment resulted in a significant increase in the MMP3, MMP9, and MMP13 protein levels, while HPG partially alleviated this trend (Figures 3D, E). The results of immunofluorescence staining further confirmed the changes in MMP13 in ECM in response to TNF-α induction and HPG treatment. The effect of HPG on TNF-α-induced ECM loss in primary NP cells was detected using AB staining. The results showed that TNF-α induced the loss of ECM in NP cells, whereas HPG protected the ECM in NP cells (Figures 4A–D).

These results suggest that HPG can counteract the increased catabolism and decreased anabolic activity of the ECM in NP cells under TNF-α-induced inflammatory stress, thereby reducing ECM loss in NP cells.

### 3.5 Effect of HPG on NP cell senescence

**FIGURE 5:**
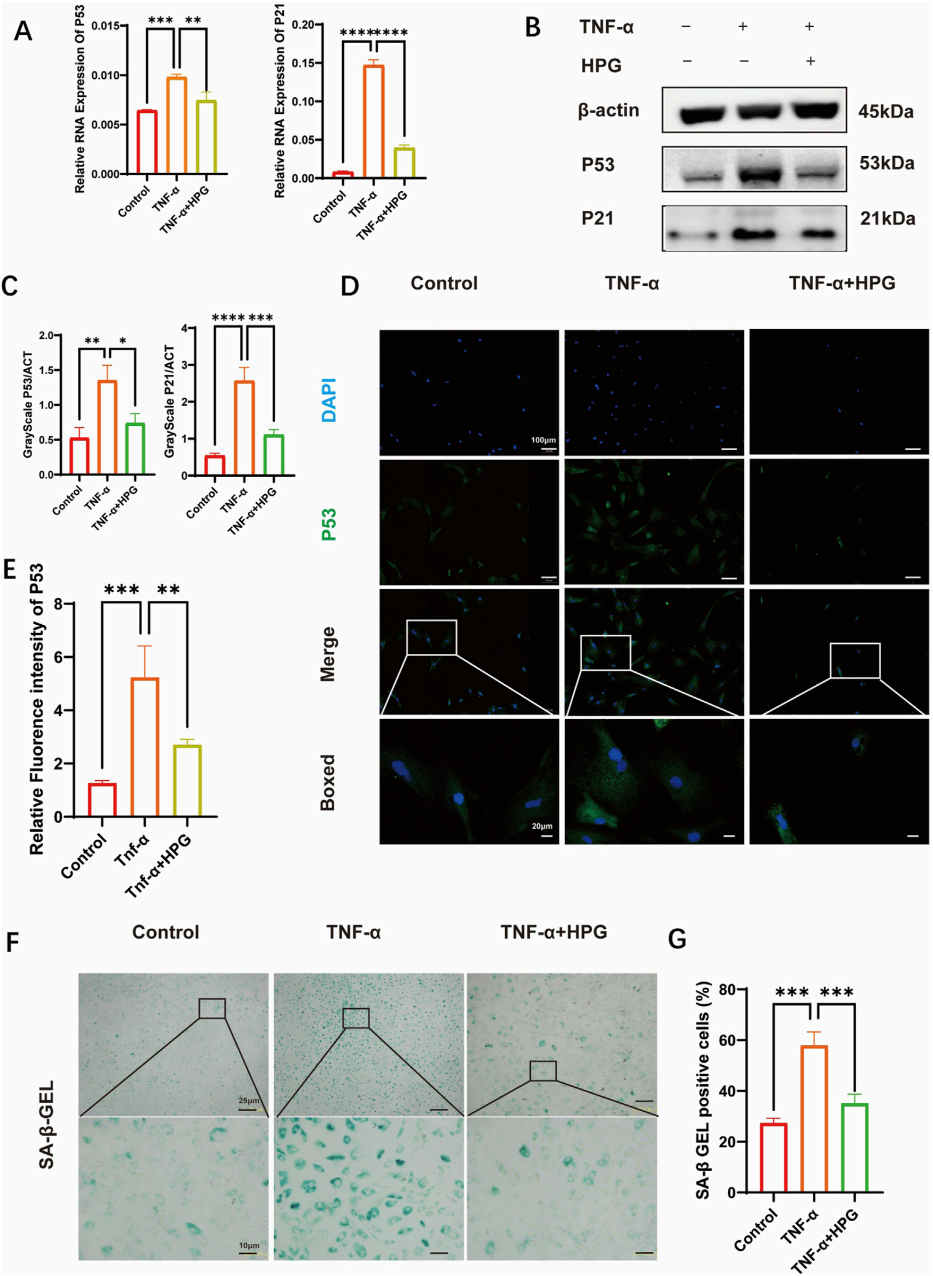
Homoplantaginin (HPG) inhibited TNF-α-induced cell senescence **(A)** The quantitative real-time polymerase chain reaction (RT-qPCR) was performed to detect the expression of the cell senescence genes after treatment with or without TNF-α (20 ng/ml) and HPG (20 μg/ml) for 24 h. The expression of those genes was normalized to the expression of β-actin (P53, P21). ALL data are presented as the mean ± SEM of three independent experiments (n = 3. **p < 0.01, ***p < 0.001 and ****p < 0.0001) **(B)** The results of western blot related to cell senescence **(C)** Gray scale analysis of western blot were perfomed to evaluate the protein expression related to cell senescence. ALL data are presented as the mean ± SEM (n = 3. *p < 0.1, **p < 0.01, ***p < 0.001 and ****p < 0.0001) **(D)** Immunofluorescence result of P53 after NP cells treated with or without TNF-α and HPG. Scale bar, 10 μm **(E)** Fluorescence intensity analysis were peformed to evaluate the expression of P53. ALL data are presented as the mean ± SEM (n = 3. **p < 0.01 and ***p < 0.001) **(F)** SA-β-Gal staining showed the senescence of NP primary cells (P3) after indicated treatment for three days. Scale bar, 10 μm **(G)** The statistics of SA-β-Gal -positive NP primary cells (P3). ALL data are presented as the mean ± SEM (n = 3. ***p < 0.001).

The effect of HPG on the expression of senescence-related genes in NP cells was detected using RT-qPCR (Figure 5A). Compared to the control group, we found that TNF-α treatment resulted in an increase in the expression of P53 and P21, which were inhibited in NP cells pretreated with HPG. As expected, we observed a consistent trend in the Western blot results. The results were also confirmed using immunofluorescence staining (Figures 5B–E). Nucleus pulposus cells were stained using a β-Gal senescence staining kit to verify the effect of HPG on NP cell senescence *in vitro*. The results showed that TNF-α treatment significantly aggravated the senescence of NP cells, which was improved by HPG treatment (Figures 5F, G). These results confirmed that HPG could counteract the TNF-α-induced senescence of- NP cells, suggesting that HPG protects against NP cell senescence and IDD.

### 3.6 Treatment of IDD with HPG *in vivo*

**FIGURE 6:**
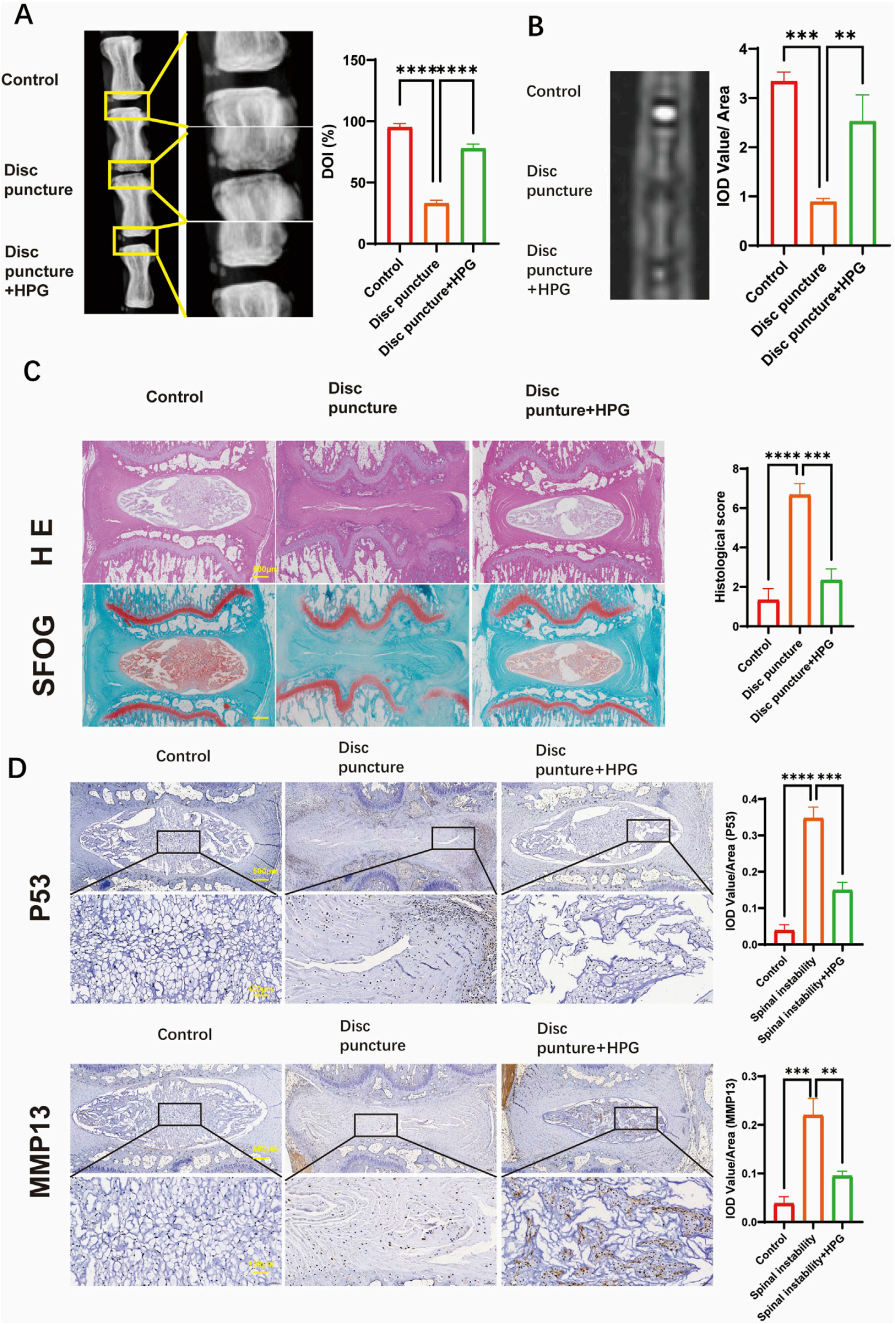
Homoplantaginin (HPG) is used to treat intervertebral disc degeneration induced by coccygeal puncture in rats in vivo **(A)** X-ray of the rat tail vertebrae one month after modeling, and quantitative analysis of disc height index (DHI, %) **(B)** MRI of the rat tail vertebrae one month after modeling and IOD Value statistics. **(C)** Hematoxylin and eosin staining nd safranin O-Fast green staining of intervertebral discs (IVDs), and Histological scores of IVDs. Scale bar, 10 μm. ALL data are presented as the mean ± SEM of three independent experiments. (n = 3. ***p < 0.001 and ****p < 0.0001) **(D)** Immunohistochemical staining of MMP13, p53 expression and their IOD Value/Area statistics. ALL data are presented as the mean ± SEM. (n = 3. **p < 0.01, ***p < 0.001 and ****p < 0.0001).

We studied the effect of HPG on IDD *in vivo* by inducing a disease model of IDD by caudal puncture in rats. Eight weeks after the caudal vertebral puncture, the caudal vertebrae of the rats were X-rayed. The results showed that the intervertebral space was significantly collapsed in the surgery group compared to the control group, reflecting the degeneration and herniation of the caudal disc, while the height of the caudal intervertebral space was maintained in the HPG treatment group(Figure 6A). Further MRI showed that the signal of the intervertebral disc in the operation group was significantly changed, indicating shrinkage and degeneration of the intervertebral disc. In contrast, local injection of HPG significantly improved the signal of the intervertebral disc(Figure 5B). Simultaneously, different groups of tissues were stained, and the degree of disc degeneration was assessed by histological scoring. The tissue scores in the HPG treatment group were significantly higher than those in the PBS injection surgery group (Figure 6C). Immunohistochemical analysis of NP tissue showed that, consistent with the *in vitro* results, the accumulation and expression of MMP13 and P53 in the NP were significantly increased in the operation group injected with PBS, while their expression was significantly inhibited in the HPG treatment group. These results indicate that local injection of HPG can prevent tail disc degeneration in rats and also suggest that local injection of HPG may be a new strategy for the nonsurgical treatment of disc degeneration (Figure 6D).

## 4 Discussion

**FIGURE 7:**
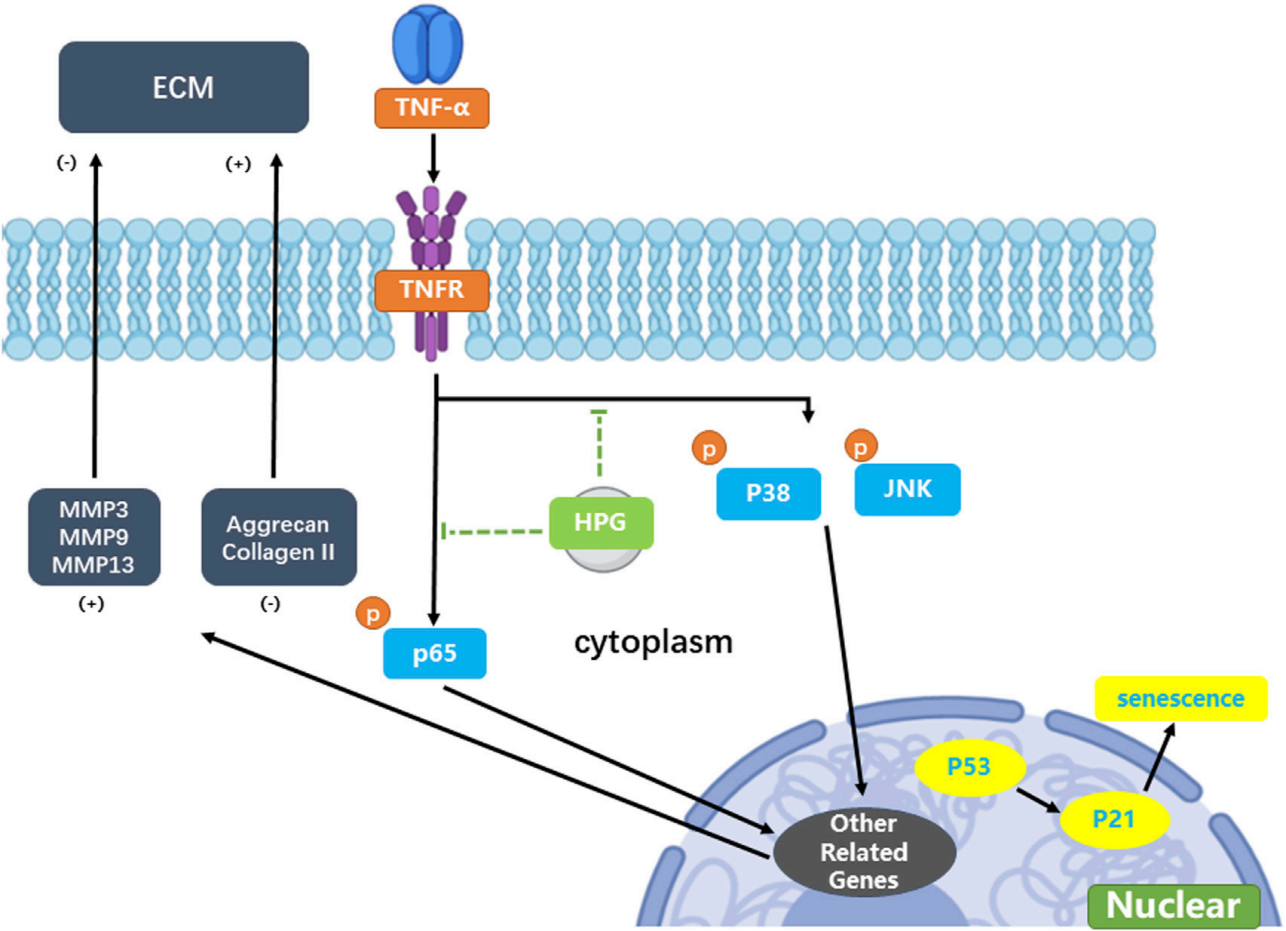
Mechanism of homoplantaginin (HPG) in tumor necrosis factor-α (TNF-α) -induced nucleus pulposus (NP) cell senescence and inflammatory response.

The annual incidence of IDD is increasing. Severe lower back pain leads to a substantial decline in the quality of life and causes great pain to the affected individuals (Chou, 2021). Lower back pain is also the number one cause of loss of working ability, which is a great burden on society (Global Burden of Disease, 2017). In recent years, owing to poor study, work, and living habits, the average age of IDD onset in the world has been decreasing. Several hospitals have reported cases of herniated discs in teenagers and children (Knezevic et al., 2021). The traditional treatment of disc degeneration focuses on surgical relief of nerve compression, fusion of adjacent vertebrae, restoration of lost intervertebral height, and pedicle screw fixation (Chou et al., 2007; Urits et al., 2019). In this process, fusion of the vertebrae can lead to a decline in flexural function (Pangarkar et al., 2019). Fused vertebrae may also lead to increased degeneration of the adjacent vertebrae (Lipson, 2004; Austevoll et al., 2021). In such cases, surgery is often not the first option for younger patients. Therefore, the search for a new alternative surgical treatment has become a hot topic in research on IDD (Figure 7). Currently, increasing attention is focused on alternatives to the surgical treatment of disc degeneration (Buser et al., 2019; Zhao et al., 2019). In current studies, IDD is generally considered to be related to oxidative stress and activation of inflammatory pathways (Khan et al., 2017; Saal, 1995). Previous studies have shown that the activation of inflammatory pathways, such as MAPK and NF-κB, upregulates the MMPs of NP cells, leading to an imbalance in the anabolism and catabolism of the ECM of the NP and further leads to the loss of ECM (Chen et al., 2021; Sun et al., 2021). With the inflammation and aging of NP cells, the NP also gradually loses water and elasticity, resulting in disc herniation, lumbar instability, and loss of intervertebral height (Kim et al., 2021). Therefore, therapy based on the inhibition of inflammatory pathway activation may be an alternative to surgical treatment for disc degeneration.

Homoplantaginin is the main flavonoid in the Chinese herb *S. miltiorrhiza*, and has anti-inflammatory and antioxidant effects (Qu et al., 2009). Homoplantaginin has been reported to inhibit TNF-α and NF-κB phosphorylation (Wu et al., 2012). In our study, the use of HPG in NP cells significantly inhibited the TNF-α-induced activation of the MAPK and NF-κB pathways, and further reduced the expression of downstream MMPs. At the cellular level, NP cells treated with HPG also showed stronger ECM secretion compared with TNF-α-stimulated NP cells alone. Our *in vivo* study further confirmed the role of HPG as an alternative therapy for IDD. In the past, the MAPK and NF-κB pathways have been reported to play an important role in disc degeneration, which is consistent with our results, and the inhibition of MAPK and NF-κB pathway activation has the expected therapeutic effect. However, inflammation is considered the source of the vast majority of diseases (Furman et al., 2019). Our study demonstrates the relationship between disc degeneration and inflammation. Furthermore, it indicates the use of HPG as an effective compound to combat inflammation.

Although the average age of onset of disc degeneration is decreasing due to poor lifestyle habits, disc degeneration was once considered an age-related disease (Bontrup et al., 2019; Iizuka et al., 2017). IDD is closely associated with aging (Antoniou et al., 1996). During aging, the intervertebral disc gradually loses water and elasticity, and inflammation accelerates this process. We also examined the TNF-α-induced senescence in NP cells and found that inflammation was associated with senescence. Accordingly, the use of HPG can also have an obvious anti-aging effect on NP cells. Compared with TNF-α alone, its combination with HPG increased the number of SA-β-Gal-positive cells and decreased the expression of P53. These findings suggest that HPG inhibits the TNF-α-induced inflammation and senescence in IDD associated with aging and inflammation. These data provide a basis for the application of HPG to the treatment of IDD.

In summary, our study demonstrates that HPG can protect NP cells by inhibiting TNF-α-induced inflammatory pathway activation and senescence, thereby maintaining the balance between the synthesis and decomposition of the NP ECM. Corresponding *in vivo* experiments further confirmed the role of HPG in the treatment of caudal disc degeneration in rats. The protective effect of HPG on IDD was observed *in vivo* and *in vitro*, confirming the anti-inflammatory effect of HPG and providing a new possibility for the nonsurgical treatment of IDD.

## Data Availability

The original contributions presented in the study are included in the article/Supplementary Material, further inquiries can be directed to the corresponding author.
