# Supplementary figures and images for: Homoplantaginin exerts therapeutic effects on intervertebral disc degeneration by alleviating TNF-α-induced nucleus pulposus cell senescence and inflammation
Source: Front Pharmacol. 2025 Mar 27;16:1526107. doi: 10.3389/fphar.2025.1526107 (PMC11983561; doi:10.3389/fphar.2025.1526107)

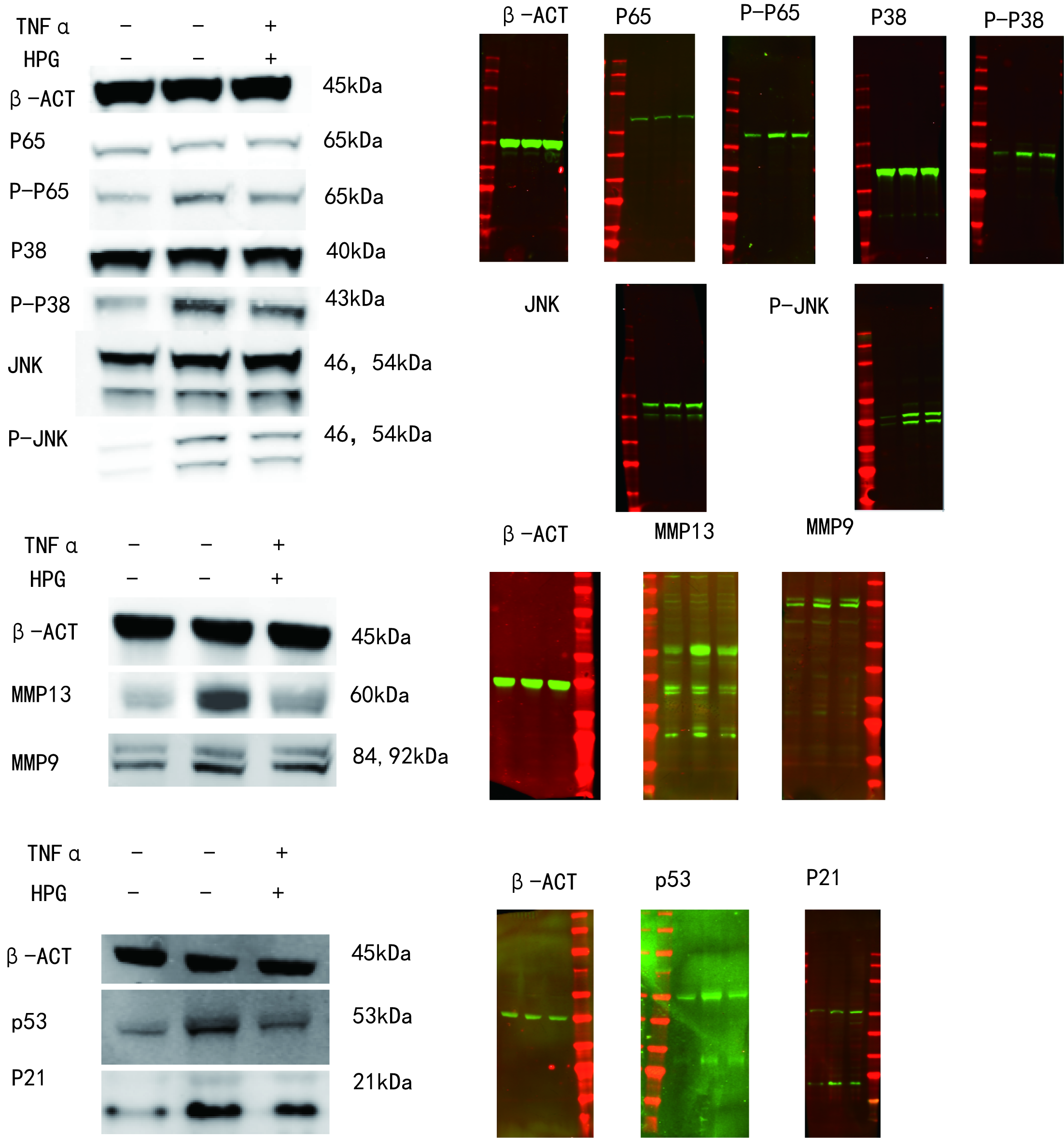

Supplement: Supplementary file 1 [file Image1.tif]
